# Vision-Based Performance Analysis of an Active Microfluidic Droplet Generation System Using Droplet Images

**DOI:** 10.3390/s22186900

**Published:** 2022-09-13

**Authors:** Amith Mudugamuwa, Samith Hettiarachchi, Gehan Melroy, Shanuka Dodampegama, Menaka Konara, Uditha Roshan, Ranjith Amarasinghe, Dumith Jayathilaka, Peihong Wang

**Affiliations:** 1 Accelerating Higher Education Expansion and Development (AHEAD) Project—Centre for Advanced Mechatronic Systems, University of Moratuwa, Katubedda 10400, Sri Lanka; 2 Department of Mechanical Engineering, University of Moratuwa, Katubedda 10400, Sri Lanka; 3 School of Physics and Materials Science, Anhui University, Hefei 230601, China

**Keywords:** active droplet generation, droplet microfluidics, performance analysis, computer vision, image processing, lab on a chip

## Abstract

This paper discusses an active droplet generation system, and the presented droplet generator successfully performs droplet generation using two fluid phases: continuous phase fluid and dispersed phase fluid. The performance of an active droplet generation system is analysed based on the droplet morphology using vision sensing and digital image processing. The proposed system in the study includes a droplet generator, camera module with image pre-processing and identification algorithm, and controller and control algorithm with a workstation computer. The overall system is able to control, sense, and analyse the generation of droplets. The main controller consists of a microcontroller, motor controller, voltage regulator, and power supply. Among the morphological features of droplets, the diameter is extracted from the images to observe the system performance. The MATLAB-based image processing algorithm consists of image acquisition, image enhancement, droplet identification, feature extraction, and analysis. RGB band filtering, thresholding, and opening are used in image pre-processing. After the image enhancement, droplet identification is performed by tracing the boundary of the droplets. The average droplet diameter varied from ~3.05 mm to ~4.04 mm in the experiments, and the average droplet diameter decrement presented a relationship of a second-order polynomial with the droplet generation time.

## 1. Introduction

**Figure 1:**
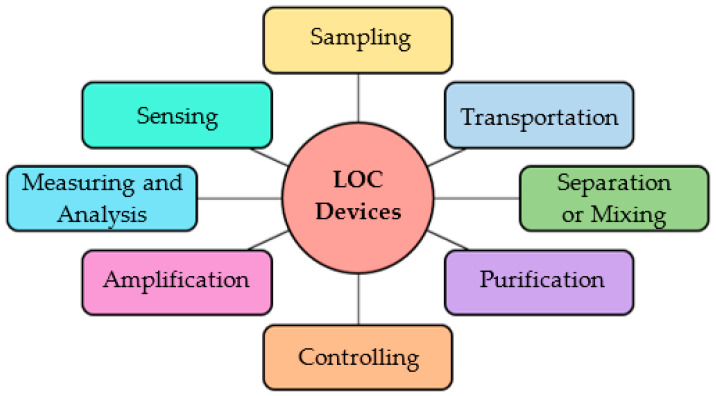
Key areas of LOC devices.

Virus and bacteria identification has been challenging yet lifesaving throughout the entire lifespan of humans. The recent COVID-19 pandemic has specified the necessity of fast, reliable, and low-cost diagnostic tools and techniques. Microelectromechanical systems (MEMS) based point-of-care (POC) devices show a promising future in satisfying the earlier requirements. Lab-on-a-chip (LOC) devices are chips developed on the micro and nanoscale, capable of performing laboratory functions on biomolecular samples such as blood, urine, sweat, and sputum. Such devices can sense, control, and actuate on the micro-scale [1]. Therefore, these devices are identified as POC devices capable of performing laboratory tests at the point of diagnostics. The literature presents comprehensive studies on the designing and development of LOC devices capable of performing various functionalities required for pathology detection, cell analysis, particle manipulation, advanced drug delivery systems, and other biomedical applications [2]. Requiring small amounts of samples and detection reagents, precise control over the cellular environment, and a higher degree of portability are the significant advantages of LOC devices [3]. Lower power demand, the minimisation of waste production, the reduction of operation cost, the capability to obtain accurate results within a short period of time, and minimising the human interactions by automating the testing process are additional benefits [4]. To perform complicated laboratory functions, the microfluidic devices require complex channel arrangements and geometries which are not feasible with the available microfabrication techniques. Small variations in microscale geometric parameters of LOC devices due to limitations imposed by microfabrication techniques, low signal-to-noise ratio due to high solvent dilutions, and interference of dominant features such as capillary forces and surface roughness with the reaction process may result in poor performance of the device [5]. Several key areas related to LOC devices identified in the literature are presented in Figure 1.

Since the biomolecular samples in the fluid phase are processed in LOC devices, microfluidic plays a major role in LOC devices. Microfluidics is a large area that deals with systematics control and manipulation of miniature fluid volumes on a micro-scale. Preliminarily, microfluidics is divided into two; continuous-flow microfluidics and droplet-based microfluidics [6]. In continuous-flow microfluidics, fluids are guided inside microchannels while maintaining the continuity of the flow. Continuous flows are used in microfluidic mixers to investigate the viability of cells (e.g., endothelial cells in different concentrations of glucose and phosphate-buffered saline) [7], segmented continuous-flow multiplex polymerase chain reaction [8], inertial microfluidics for bioparticle focusing and separation [9], and to perform microfluidic sieving to separate target red-fluorescent proteins in protein mixtures [10]. Droplet-based microfluidics deals with generating and manipulating discrete minute-scale fluid volumes through immiscible multiphase flows inside microchannels. It has advantages in miniaturisation, compartmentalisation, and parallelisation [11]. Droplet-based microfluidics is dominant in detecting molecules with a higher sensitivity and throughput in single-molecule experiments (e.g., fluorescence polarisation immunoassay to determine angiogenin concentration) [12], developing a microfluidic platform to identify bacteria (e.g., Escherichia Coli in water) [13], cell biology to produce artificial lipid bilayers [14], and to engineer polymeric microcapsules [15]. Droplet generation is essential in droplet microfluidic systems where precise sample preparation and controlled transportation are required. Generally, the droplet generation consists of four stages; lagging, filling, necking, and detachment [16]. Common methods studied for droplet generation are geometry-based, aspiration-based, slip-chip and digital microfluidic methods such as electrowetting, dielectrophoresis, opto-electrowetting, thermos-capillary effect and surface acoustic waves [17,18,19]. The geometry-based method utilises the geometric arrangement of the microchannels to generate droplets. Cross-flow, flow-focusing, and co-flow are three geometric arrangements widely used in the literature [20]. The droplet generators are categorised into two classes: active and passive devices. Active devices use external forces to drive the flow, and no external forces are used in passive devices. Comparatively, active devices provide higher controllability because of the ability to regulate using external forces precisely [21].

Among the various detection techniques used on the macro-scale, several mechanical, chemical, optical, electrochemical, and nanomaterials-based techniques are applicable on the micro-scale to perform object identification and feature extraction in LOC systems [22]. The wide range of applications has resulted in a spectacular technological advancement of these techniques. Optical sensing is a technique in which an electromagnetic wave or a change of electromagnetic wave is converted to an electrical signal to acquire data [23]. Optical sensing is widely applied in microfluidic research because of the ability to sense in real-time and the higher degree of accessibility to required hardware such as microscopes, cameras (both CCD and CMOS), and most recently, smartphones. The simplicity of interfacing such hardware together with microfluidic systems has enabled methods such as fluorescence detection, colourimetric detection, chemiluminescence detection, interferometric detection, and surface plasmon resonance-based detection [24]. The most common method, fluorescence detection, is based on the absorption and emission of light energy from the particles under investigation, and this method is utilised as a real-time and endpoint sensing technique. Recently, fluorescence detection-based POC devices integrated with internet of things (IoT) technology to diagnose infectious diseases and monitor their spread have been reported [25]. In addition, quantum dot-based sensing techniques have been used to develop paper-based analytical devices, and these nanoparticles allow for the accurate tracking of bioparticles [26]. Vision sensing is a branch of optical sensing which involve data acquisition using a camera module, data processing, and analysis of the captured image. The advancements in electronics, manufacturing, and computational ability have enabled vision sensing applicable for miniaturised systems, such as microfluidic devices [27]. Vision systems and algorithms can perform multiplex operations accurately with a fast response time and high sensitivity and acquire detailed data with minimum hardware interactions with other components in a non-contact manner [28]. Digital Image Processing (DIP) techniques can extract single or multiple features using a digital image [29], which is highly beneficial in object identification. Hence, it is required to perform complex data analysis procedures to analyse the acquired information [30]. DIP is performed at several stages: image acquisition, enhancement, segmentation, feature extraction, and analysis using appropriate techniques suitable for the applications. Various algorithms to perform morphological analysis on droplet images are reported in the literature [16]. Droplet generation systems are characterised using the features of the generated droplets. The droplet size is most commonly considered the governing parameter in analysing a droplet generation system’s performance [31]. Table 1 presents features of objects which are identified in droplet images using DIP.

Precise droplet production and manipulation are critical in droplet-based bioassays. Droplet-based analytical devices have demonstrated promising results, but most systems are trained to generate predefined droplets, and the devices are incapable of adapting to performing complex bioassays which require step-by-step processing. On-demand droplet generation systems are important in this regard for applications which specifically deal with extremely dilute samples [48]. Previously discussed on-demand droplet generation systems are developed using expensive hardware and software which are not accessible in general-purpose laboratories. In this study, we investigated a low-cost system feasible with a microfluidic droplet generator fabricated using a laser-based fabrication technique, an in-house developed controller, an experimental setup with fluidic and electrical connections, and control software for the performance analysis using DIP. We have conducted an experimental performance analysis using the droplet size on a geometry-based active droplet generator to understand the system’s behaviour. A powerful technique, vision-based non-contact sensing followed by DIP, is used for data acquisition and processing. In a geometry-based microfluidic droplet generator, the geometric parameters of the device significantly affect the droplet generation process [49]. Therefore, it is of utmost importance to study the behaviour of the droplet generation system, specifically the variation of droplet size with time. Based on that study, the droplet generation system can be characterised and further investigate the suitability for on-demand droplet generation.

## 2. Materials and Methods

### 2.1. Proposed System

**Figure 2:**
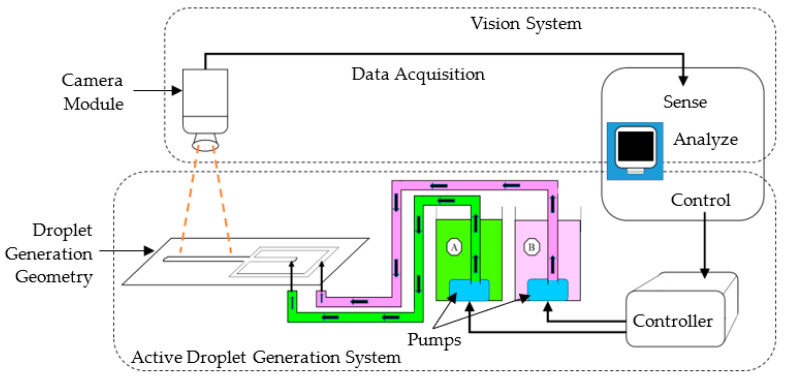
Proposed system.

As shown in Figure 2, the proposed hardware configuration consisted of a droplet generation geometry, two reservoirs, two submersible pumps, fluid supply tubes, a controller, a camera, and a workstation computer.

#### 2.1.1. Droplet Generator

**Figure 3:**
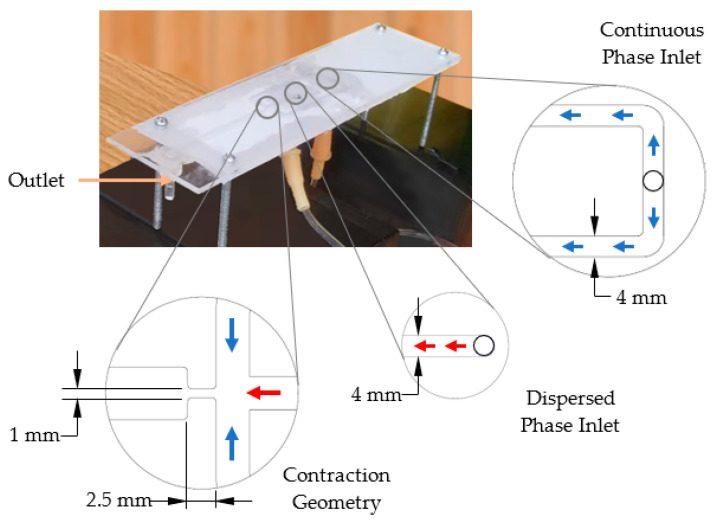
Droplet generator.

The droplet generator used in this study was based on flow-focusing geometry, and the device was previously developed by the research group [50]. The laser machining technique was used to fabricate the layers of the design, and polymethyl methacrylate (PMMA) was used as the material. Finally, the layers were assembled using a thermal bonding process. As shown in Figure 3, the droplet generator included two inlets, one for each fluid phase and an outlet channel having a width of 6 mm to guide the fluid flow for further observation and analysis. As illustrated in the inset image, the height of inlet channels, outlet channel, and contraction geometry was 1 mm. The contraction geometry was designed with a reduction of the channel width, and it directly affected the droplet formation process. All of the dimensions were obtained using numerical simulations [49,50].

#### 2.1.2. Controller and Control Algorithm

**Figure 4:**
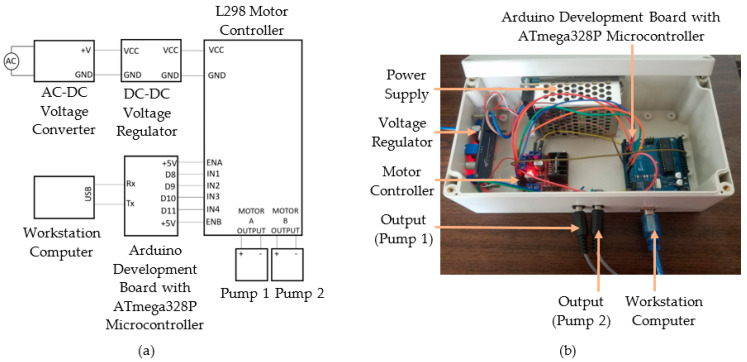
Controller for the active droplet generation: (**a**) Wiring diagram; (**b**) Assembled device.

The controller consisted of a microcontroller, dual-channel H-bridge motor driver, voltage regulator, and a power supply. The control algorithm was deployed in a workstation computer to control the system. The control signal was transmitted to the microcontroller, and the subsequent output signal was transmitted to the submersible pumps through the motor controller outputs. Figure 4a shows the wiring diagram of the controller, whereas Figure 4b represents the assembled controller.

ATmega328P-based microcontroller drove the direct current (DC) motors inside the submersible pumps. The dual-channel H-bridge motor driver drove the DC motors, and the motor driver controlled the motor speed. A power supply with an AC-DC converter was selected to obtain a 12 V DC supply from a 230 V input. The voltage regulator modulated 12 V DC input in a range from 1.1 V to 12 V. Two centrifugal-type submersible pumps were placed inside the reservoirs. Two pumps must be independently controlled to supply continuous phase and dispersed phase fluids at different flow rates, which was essential in obtaining the required flow rate ratios for droplet generation. MATLAB software with the Arduino hardware support package [51] was used to deploy the control algorithm to receive user inputs and transmit the control signal to the microcontroller. The microcontroller generated the pulse width modulation (PWM) signal to control the motor controller. The output signals of the motor controller were generated separately at two output channels to regulate the flow rate of the pumps resulting in a controlled fluid flow towards the droplet generator.

### 2.2. System Architecture

**Figure 5:**
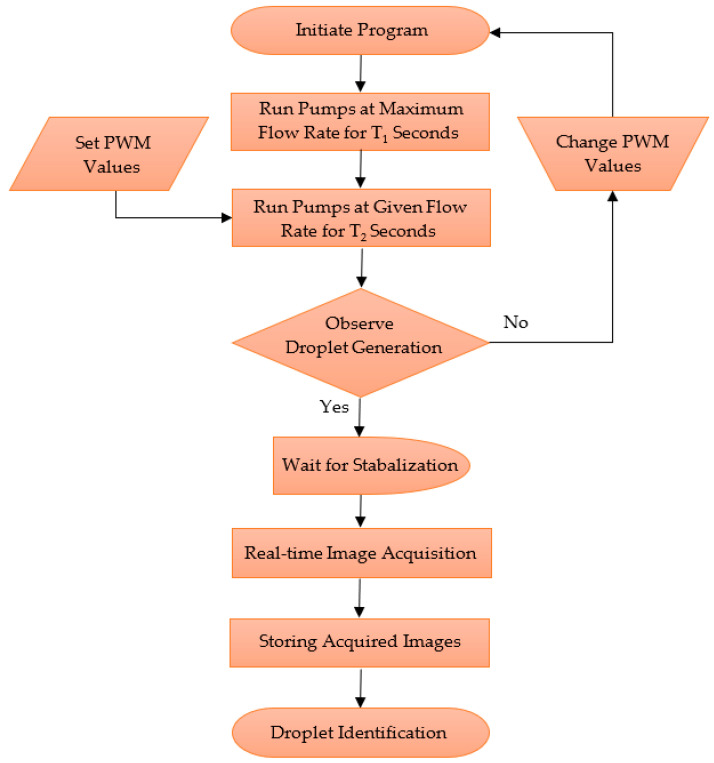
Control architecture of the system.

The proposed system architecture for the study’s performance analysis consisted of steps belonging to the two major subsystems (shown in Figure 2); the active droplet generation and vision systems. Figure 5 shows the steps of controlling the active droplet generation and vision-based droplet identification systems.

As explained previously, the droplet generation system was deployed based on the PWM values. The vision system was deployed based on time, and it was initiated after an interval to provide an adequate time for the system to stabilise. The captured images were stored in the internal storage of the workstation computer, and then the images were processed to analyse the performance of the active droplet generation system.

### 2.3. Vision-Based Droplet Identification and Feature Extraction

Image acquisition was integrated with the control algorithm of the active droplet generation system. Therefore, once the droplet generation was initiated, the algorithm could acquire images in real-time and store them throughout a given interval. The images were captured with a resolution of 640 × 480 using a CMOS-based camera module mounted above the output channel of the droplet generator. As a result, the images were captured from the top view.

#### 2.3.1. Pre-Processing Droplet Images

The droplet identification algorithm used several filtering and thresholding steps to isolate and differentiate droplets by reducing background noises. Band filtering, binary conversion, thresholding, and opening techniques were used. Band filtering is based on extracting red, green, and blue colour bands from an RGB image to enhance the features in the image. Different band images assist in object identification depending on the background colour and the object colour. Then, the band image was converted to a binary form: a matrix having values between 1 and 0 depending on the brightness. Assuming the resolution of the images captured using the camera is M × N, the RGB format of the image was represented by a three-dimensional matrix of size M × N × 3. The third dimension represents details of the image’s red, green, and blue segments. The three colour layers of the RGB images were extracted to identify the suitable band image for further processing. Then, the band image was converted to a binary image. Equation (1) defines the threshold method:

(1) $$BI\left(x,y\right)=\{\;\;\begin{array}{c} RBI\left(x,y\right)\geq ThrV\;\;\;\;\;\;1\\ RBI\left(x,y\right)<ThrV\;\;\;\;\;\;0 \end{array}$$

*RBI* was the band image which was the input image. The pixel position was represented using *x* and *y* coordinates towards the row and column directions. *ThrV* was the thresholding value, and *BI* was the binary image generated by the binarization algorithm. A threshold value was required to separate pixels that belonged to the droplets and the background. In image processing, morphological operations such as erosion, dilation, opening, and closing provided the capability of removing imperfections in the input images [52]. In this study, the opening operation, which is a combination of erosion and dilation, was used to remove the noises, and Equation (2) describes the opening of an input image (*A*) by a structuring element (*B*):

(2) A ο B=(A ⊖B) ⊕B

#### 2.3.2. Identification of Droplet Boundary

Moore-neighbourhood and von Neumann neighbourhood are the two most common approaches based on cellular automata to define the neighbourhood for boundary tracing [53]. In this study, the Moore-neighbour tracing algorithm (MNTA) followed by Jacob’s stopping criterion was implemented to trace the boundary of each droplet [54]. Moore-neighbourhood (*N^M^*) of range ‘*r*’ for a set of pixels surrounding a given pixel at (*x_0_*, *y_0_*) coordinates is given by the Equation (3):

(3) $$N_{\left(x_{0},y_{0}\right)}^{M}=\left\{\;\left(x,y\right)\;:\;\left|x-x_{0}\right|\;\leq r,\;\;\;\left|y-y_{0}\right|\;\leq r\;\right\}$$

In MATLAB, MNTA is deployed based on 8 connected pixels surrounding a given pixel (P). In identifying the boundary of a droplet, MNTA scans the Moore-neighbourhood searching for a white pixel. Initially, the image segment was scanned in sequential order until a white pixel was found. After a white pixel was found in the Moore-neighbourhood, that white pixel was set as the pixel P. The scanning process restarted on the newly defined Moore-neighbourhood surrounding the pixel P. The stopping criterion played a significant role in object identification. Jacob’s stopping criterion was used in this work. In Jacob’s stopping criterion, the scanning algorithm is terminated after entering the starting pixel a second time in the same way it initially entered.

#### 2.3.3. Extracting Droplet Features for Performance Analysis

Droplet diameter is the morphological feature that was studied in this research. Area and perimeter values were used to calculate the average diameter to test the system’s performance. In this regard, the areas of the identified droplets were obtained by counting the number of white pixels that belong to each droplet separately. The perimeter was also obtained in pixels. The radius of each droplet in an image was calculated in pixels using the obtained area and the perimeter separately. The average radius of each droplet was calculated using the two radius values. The radii values of the droplets in an image were averaged, and an average droplet diameter value was defined for each image. Finally, the average droplet diameter values were converted to millimetres by defining the pixel-to-millimetre ratio based on the outlet channel width (6 mm), which was a known parameter.

### 2.4. Experimental Setup for Vision-Based Droplet Generation System

**Figure 6:**
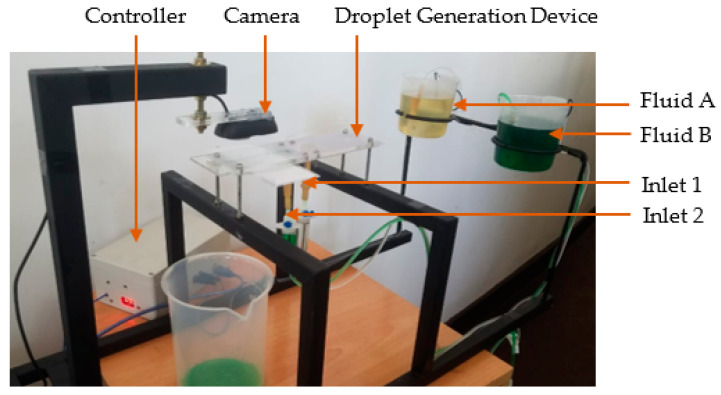
Experimental setup for vision-based droplet generation system.

The hardware arrangement used for the experiment is shown in Figure 6. Submersible pumps were attached to each reservoir’s bottom surface, containing continuous phase (fluid A) and dispersed phase (fluid B). The submersible pumps used in the experimental setup had a voltage rating of 9 V. However, the voltage regulator was set to 9.4 V to compensate for the voltage drop in the voltage regulator. Therefore, the PWM value varied for the voltage range from 0 to 9 V with a resolution of 0.035 V.

For the experiment, coconut oil having 900 kgm^−3^ density and 55 cP viscosity was used as the continuous phase, and water having 1000 kgm^−3^ density and 1.01 cP viscosity was used as the dispersed phase. The coloured water was used as the colour difference increased the capability to differentiate the water droplets from coconut oil flow in image processing. Since the study was based on the variation of droplet diameters at a given period, the setup was configured to generate droplets for a fixed time period and capture images.

### 2.5. Design and Development of Graphical User Interface (GUI) for Vision-Based Performance Analysis

**Figure 7:**
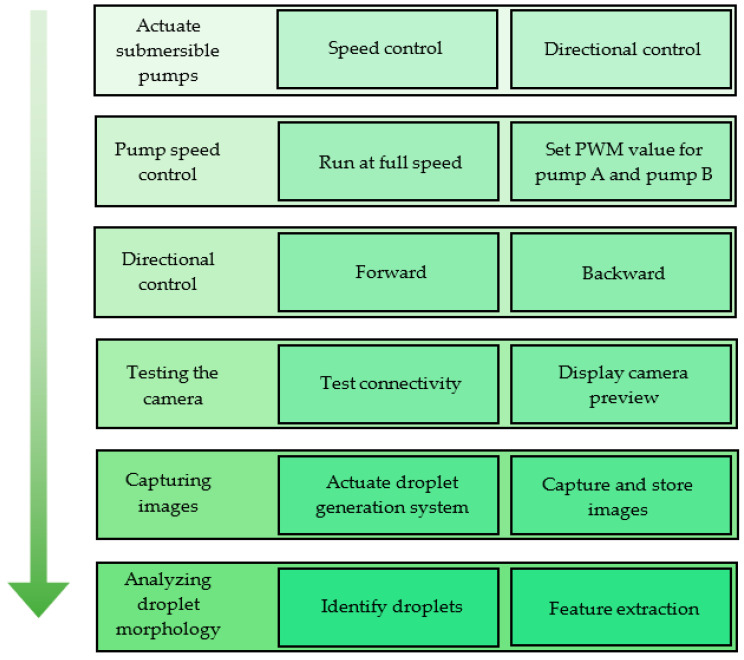
Functionalities of the system and features of the GUI.

The functional requirements of the overall setup and the related features in the developed software are shown in Figure 7.

**Figure 8:**
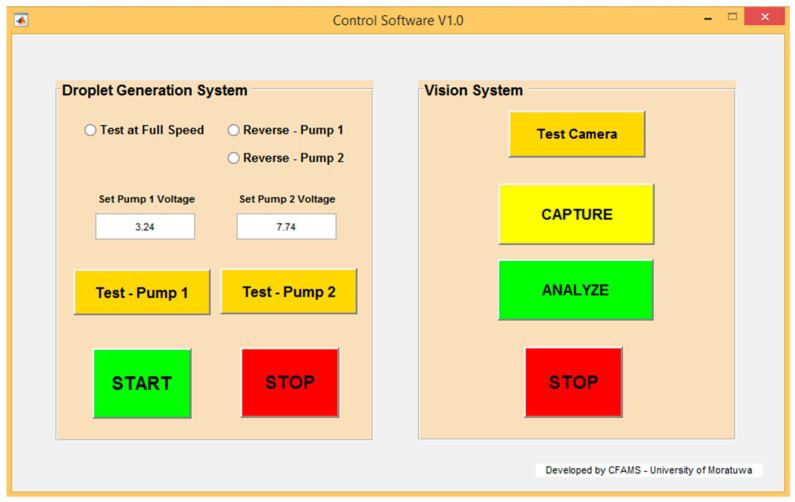
Front-end of the GUI.

MATLAB GUI development environment (GUIDE) was used to develop the GUI. The GUI designed for the experiment is shown in Figure 8, and it was developed with features to test the droplet generation system separately. Therefore, droplet generation can be monitored without running an entire experiment. Using the GUI, the pumps could be tested at full speed or a given PWM value. Additionally, it was possible to reverse the fluid flow, which allowed the system to remove the fluid inside the channels and the droplet generator to reduce the stagnation of debris in the device, as it caused errors in experiments.

## 3. Results and Discussion

**Figure 9:**
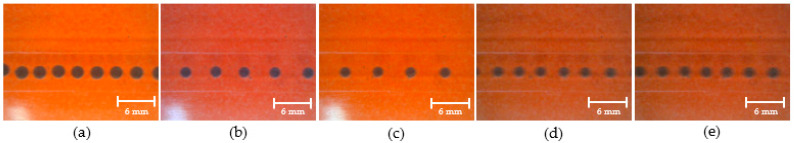
Visible variation of brightness levels: (**a**) t = 15 s; (**b**) t = 615 s; (**c**) t = 1215 s; (**d**) t = 1815 s; (**e**) t = 2415 s.

At first, a droplet generation experiment was carried out for 45 min to observe the camera’s performance, the effect of the lighting in the laboratory environment, and the quality of the acquired images. Starting at t = 15 s, the images were captured at 15 s intervals. Figure 9 shows images captured at 10 min intervals.

The resulting images (as shown in Figure 9) convey a visible brightness variation when the test was carried out over a long period. The lighting conditions affected the captured images and provided faulty results for a fixed threshold value in image processing. Therefore, the surrounding lighting conditions were identified as a significant parameter, and with that consideration, it was carefully controlled to acquire the experimental images.

### 3.1. Detecting Droplets in a Single Image

**Figure 10:**
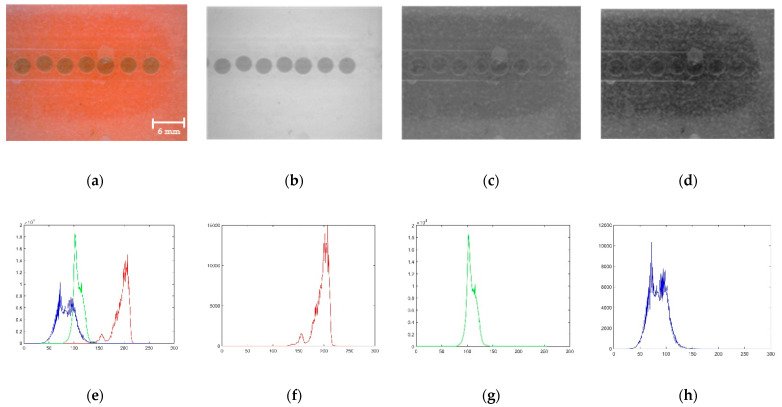
Band filtering: (**a**) Original image; (**b**) Red segment; (**c**) Green segment; (**d**) Blue segment; (**e**–**h**) Respective histograms.

The three colour layers of the RGB image are presented as the third dimension of the M × N × 3 matrices. The original RGB image is shown in Figure 10a, and the extracted red, green, and blue band segments are shown in Figure 10b–d. Respective histograms are shown in Figure 10e–h. The red band segment of the RGB image is selected to proceed with further analysis by observing the band images because the red band segment displays a clear difference between the pixels that belong to droplets and the background.

**Figure 11:**
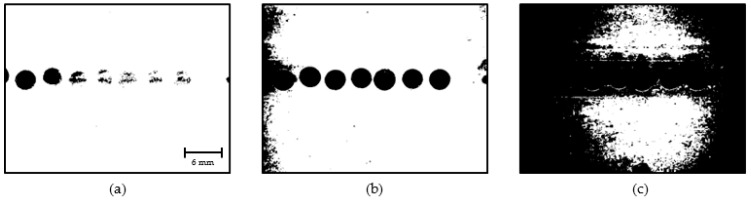
Comparison of threshold values: (**a**) 0.6; (**b**) 0.7; (**c**) 0.8.

In binarization, each pixel’s grey value in the image is converted to a value between 0 and 1. With this, 0 represents black pixels, and 1 represents white pixels. The threshold value is varied from 0.1 to 0.9 to identify the optimum thresholding value. As shown in Figure 11, traces of droplets are visible in the range from 0.6 to 0.8, and hence the optimum thresholding value is identified as 0.7.

**Figure 12:**
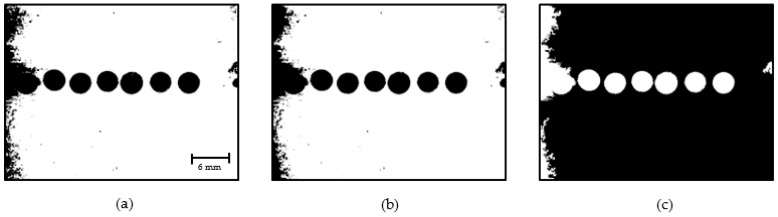
Morphological opening: (**a**) Binary image; (**b**) Opening step 1; (**c**) Opening step 2.

The obtained binary images of the droplets presented with noise caused due to lighting conditions, tiny water particles in the coconut oil flow, stagnated fluid particles near the channel walls, and uneven surface properties in the channel walls. Such noises are identified as small objects and are filtered based on their size. Figure 12 shows the stages of the opening operation, which reduces unnecessary noises present in binary images.

**Figure 13:**
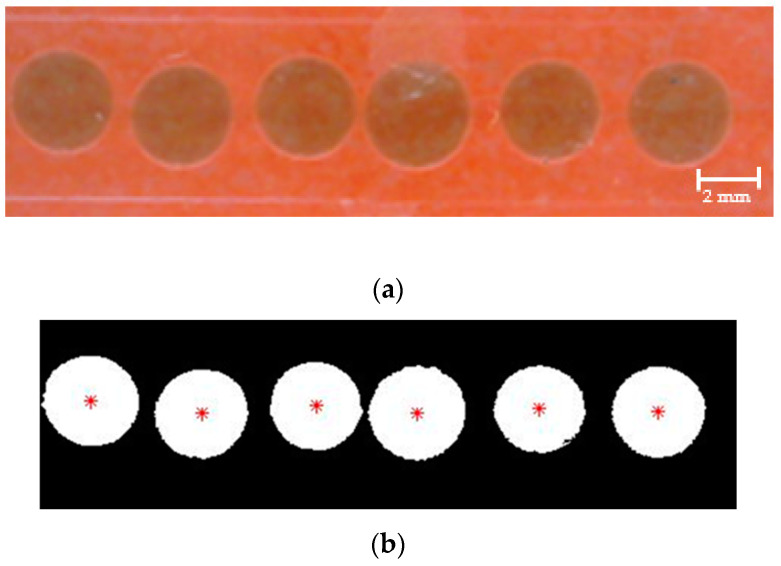
Droplet identification: (**a**) Original RGB image; (**b**) Detected droplets using DIP.

As shown in Figure 12c, the droplet area is visible; therefore, the droplets are identified by detecting and tracing the external boundary of the droplets. A region that includes a segment of the outlet channel of the droplet generator is considered for droplet identification, as shown in Figure 13. The pixels belonging to droplets are represented in white, and the background pixels are black.

### 3.2. Performance Analysis

**Figure 14:**
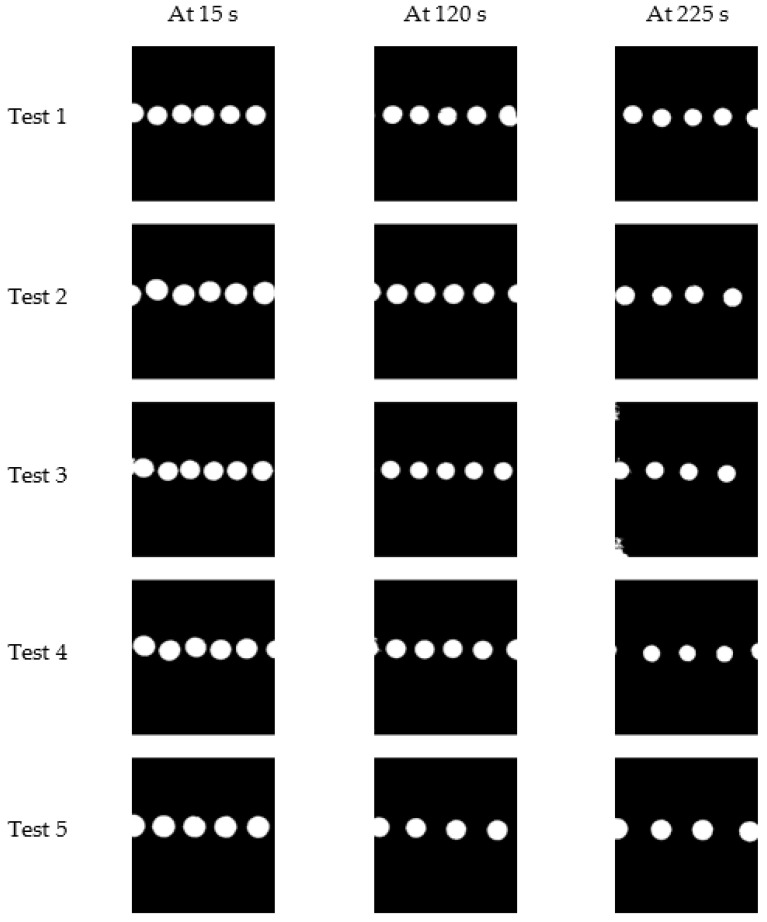
First, middle, and last image of each experiment.

The tests were conducted to observe the system’s behaviour and analyse its performance for 225 s each at 15 s intervals, and five image sets were selected to process further. Figure 14 shows the first (at 15 s), middle (at 120 s), and the last (at 225 s) image of each test after the image pre-processing steps.

**Figure 15:**
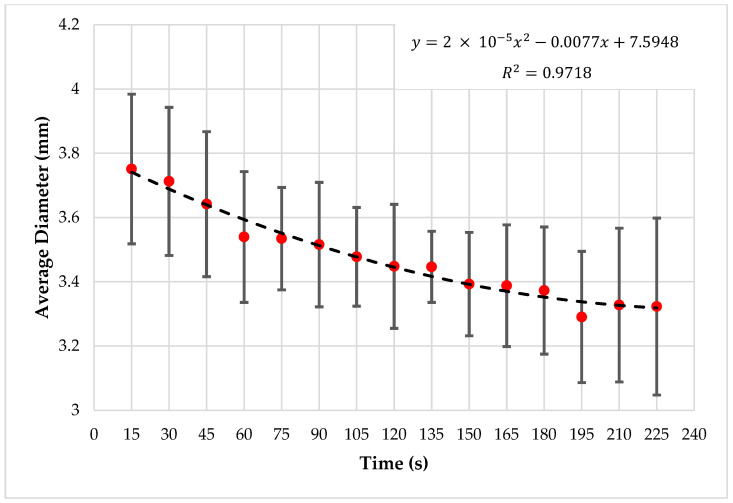
Time vs. average droplet diameter.

It was observed that there is a decrement in droplet diameter with time, and the droplet generation frequency is also decreasing, as shown in Figure 14. Droplet detection and feature extraction were done for all the 75 images in five image sets to obtain the area and perimeter of droplets in each image. The average droplet diameter of each image in a test was observed to have a decrement throughout the experimenting period, and Figure 15 is plotted considering the average of the tests.

The graph shows that the diameter of the generated droplets decreased with the experiment time in all the tests, and the decrement equates to a second-order polynomial. In addition, the average droplet diameter is varied in a range from ~3.05 mm to ~4.04 mm.

## 4. Conclusions

The active droplet generation system’s performance was considered in this study, and the presented experimental setup successfully generated droplets based on the active flow-focusing method having two submersible pumps. These two pumps were controlled separately to obtain the required flow rate ratios for droplet generation. The control signals were provided from a GUI to the ATmega328P-based controller. Image processing was used to process captured images and to calculate the droplet diameter over five experiments using a total number of 75 images. Band filtering, binarization, thresholding, and opening techniques eliminated the background noises and isolated the droplets in an image. The red band segment produced during band filtering was used as the input image for binarization.

Furthermore, an optimum threshold value (ThrV) of 0.7 was identified experimentally and used in the binarization algorithm. Boundaries of droplets were traced using Moore-neighbor tracing algorithm followed by Jacob’s stopping criterion. The area and perimeter of droplets were obtained in pixels to calculate an average droplet diameter in pixels. Then, the average droplet diameter values were converted to millimetres at a ratio of 17.128 pixels per millimetre, which is obtained using the ‘ImageJ’ software. In the performance analysis of the droplet generator, an average droplet diameter value defined for each image and droplet generation frequency had been observed. A decrement in droplet diameter and droplet generation frequency was experienced with time.

According to the results obtained using the aforementioned experimental setup, it was realised that several other factors affect the droplet diameter and the generation frequency. The flow rate was not constant even under a fixed voltage supply. In addition, the effect of the reservoir head was not negligible as the flow rate depends on the reservoir head. The channel surface chemistry, geometry of the microfluidic chip, flow actuation stability, and total-flow rate ratio significantly affect the uniform and stable droplet generation. Further studies on controlling the reservoir head and precise microfluidic pumping systems are required to improve this system to generate uniform droplets at a constant frequency for extended periods. In addition to that, high-end vision hardware and AI-based feature extraction and analysis will help to achieve better performance.

## Figures and Tables

**Table 1:** Identifiable features using DIP techniques.

Feature Identification					
Size [32]	Eccentricity [33]	Gradient [34]	Concavity [35]	Projected Surface Area [36]	Sphericity Index [37]
Symmetry [38]	Roundness [39]	Border [40]	Radius [41]	Membrane Surface Area [42]	Contrast Variations [30]
Shape [43]	Elongation [44]	Saturation [45]	Volume [38]	Sphericity Coefficient [46]	Form factor [47]
